# An overview of Uganda's mental health care system: results from an assessment using the world health organization's assessment instrument for mental health systems (WHO-AIMS)

**DOI:** 10.1186/1752-4458-4-1

**Published:** 2010-01-20

**Authors:** Fred Kigozi, Joshua Ssebunnya, Dorothy Kizza, Sara Cooper, Sheila Ndyanabangi, the Mental Health and Poverty Project

**Affiliations:** 1 Butabika National Referral and Teaching Mental Hospital, Kampala, Uganda; 2 Department of Mental Health and Community Psychology, Makerere University, Kampala, Uganda; 3 Department of Psychiatry and Mental Health, University of Cape Town, South Africa; 4 Mental Health Department, Ministry of Health Headquarters, Kampala, Uganda

## Abstract

**Background:**

The Ugandan government recognizes mental health as a serious public health and development concern, and has of recent implemented a number of reforms aimed at strengthening the country's mental health system. The aim of this study was to provide a profile of the current mental health policy, legislation and services in Uganda.

**Methods:**

A survey was conducted of public sector mental health policy and legislation, and service resources and utilisation in Uganda, in the year 2005, using the World Health Organization's Assessment Instrument for Mental Health Systems (WHO-AIMS) Version 2.2.

**Results:**

Uganda's draft mental health policy encompasses many positive reforms, including decentralization and integration of mental health services into Primary Health Care (PHC). The mental health legislation is however outdated and offensive. Services are still significantly underfunded (with only 1% of the health expenditure going to mental health), and skewed towards urban areas. Per 100,000 population, there were 1.83 beds in mental hospitals, 1.4 beds in community based psychiatric inpatient units, and 0.42 beds in forensic facilities. The total personnel working in mental health facilities were 310 (1.13 per 100,000 population). Only 0.8% of the medical doctors and 4% of the nurses had specialized in psychiatry.

**Conclusion:**

Although there have been important developments in Uganda's mental health policy and services, there remains a number of shortcomings, especially in terms of resources and service delivery. There is an urgent need for more research on the current burden of mental disorders and the functioning of mental health programs and services in Uganda.

## Introduction

Uganda is a landlocked country located in East Africa with an approximate geographical area of 236,040 square kilometers, of which about 15.4% is covered by water. As of 2006, the population of Uganda was estimated to be 27.4 million people, having increased from 24.2 million in 2002 at a growth rate of 3.3%. Of these, 48.6% were males and 51.4% females. An estimated 12.3% of the population were living in the urban areas while 87.7% were living in the rural areas. The life expectancy at birth for males was 50.7 and 52.7 for females, while the literacy rate for men was 76% and 61% for women [1,2]. English is the official language taught in schools, used in courts of law, most Newspapers and radio broadcasts; while Luganda is the most widely used native language. According to the 2002 National Population and Housing Census, the population is predominantly Christian, composed of: Roman Catholics (41.9%), Protestants (35.9%), Muslims (12.1%) and others (10.1%) [3].

Based on World Bank criteria [4], Uganda is a low-income country. The average per capita income in Uganda is USD 300, with 31% of the population living below the poverty line [5]. This economic situation has implications for mental health in Uganda, given emerging evidence from low-income countries that mental illness and poverty have a dialectical relation, reinforcing and exacerbating each other [6-9].

The Ugandan government has recently realized that mental health is a serious public health and development issue. Consequently, the Ugandan mental health program was initiated in 1996, and subsequently strengthened by the launch of the National Policy and Health Sector Strategic Plan in 1999/2000. Mental health is now included as one of twelve components of the National Minimum Health Care Package, to be provided at all levels of care [10,11].

Despite the reforms and subsequent improvement of mental health services, Uganda's mental health system still faces a number of shortcomings. The current Mental Health Act, last revised in 1964, is outdated and offensive [12]. Furthermore, there is a general lack of trained human resources and a scarcity of funding, with no special provisions for mental health funding in the draft mental health policy [13].

This report is on the public sector mental health system in Uganda, and provides a comprehensive overview of the current mental health policy, legislation and services in Uganda. The paper presents the quantitative results of a situation analysis of the mental health system in Uganda, which was conducted as part of the first phase of the Mental Health and Poverty Project (MHaPP). The MHaPP, which is being conducted in four African countries: Ghana, South Africa, Uganda and Zambia, aims to investigate the policy level interventions required to break the vicious cycle of human poverty and mental ill-health, in order to generate lessons for a range of low- and middle-income countries [6].

## Methods

An assessment of the mental health system in Uganda in the year 2005 was conducted using the World Health Organization's Assessment Instrument for Mental Health Systems (WHO-AIMS) Version 2.2 [14]. The WHO-AIMS tool has been developed to assess key components of a mental health system, thereby providing essential information to strengthen mental health systems. The instrument was developed following the publication of the World Health Report 2001 [15], which focused on mental health and covers the 10 recommendations made in this report. It consists of 6 domains 28 facets and 156 items. The 6 domains are interdependent, conceptually interlinked, and somewhat overlapping. The domains include:

• Domain 1: Policy and legislative framework

• Domain 2: Mental health services

• Domain 3: Mental health in primary care

• Domain 4: Human resources

• Domain 5: Public education and links with other sectors

• Domain 6: Monitoring and research.

Shorter questionnaires seeking specific information were generated directly from the 156 items in the WHO-AIMS document. These shorter questionnaires were distributed to specific resourceful persons believed to have the required information, by virtue of their positions. The respondents were from the following institutions:

• Mental health division, Ministry of Health headquarters

• Department of psychiatry, Makerere University Medical School

• Makerere University Institute of Psychology

• Mental Health professionals and the records office at the National Mental Hospital

• Uganda Nurses and Midwives Council

• Mental Health non-government organizations (NGOs) and User associations

The purpose of the study was to document all public sector mental health service provision in Uganda. Private-for-profit services were not included as they only provide services for a minority of the population, and are seldom utilised by those who live in conditions of poverty [16].

Data were collected in 2006, based on the calendar year 2005. The data were entered into the excel data entry programme where numeric data were aggregated. Descriptive statistical analyses of relevant items were conducted. Permission to conduct this study was obtained from the National Council for Research, and the Director General of Health Services

## Results

### Mental health policy and plans

The mental health programme at the Ugandan Ministry of Health was initiated in 1996, for coordination of mental health services in the country. Existence of a public health and primary health care framework for the achievement of the health care goals facilitated the inclusion and integration of mental health into Primary Health Care (PHC). In accordance with the health sector reforms and plans, a draft mental health policy was developed in 2000, and still remained a draft by the year 2006. The following WHO-AIMS items/components are addressed in this draft mental health policy:

• Developing community mental health services

• Decentralization of mental health services

• Integration of mental health services into Primary Health Care.

• Human resources

• Involvement of users and families

• Advocacy, education and promotion of mental health

• Human rights protection of users

• Equity of access to mental health services across different groups

• A monitoring system

The WHO-AIMS items/components not addressed in this draft mental health policy are:

• Financing for mental health activities

• Quality improvement

• The relationship between mental illness and poverty

• The role of allied health service providers such as psychologists and social workers

• Provision for welfare benefits for people with mental illness

• Research and policy evaluation

• Child and adolescent mental health issues are mentioned, but not strongly addressed.

• Issues of conflict and mental health

Despites these gaps the draft policy has informed service reforms within the country, which have made significant strides towards strengthening mental health services in the country. These include decentralization of mental health services; integration of mental health into PHC up to the community level, construction of mental health inpatient units within the Regional Referral Hospitals; training of staff at all levels (pre-service and in-service training) and the involvement of other players such as Civil Society Organizations, Traditional healers, and other relevant sectors.

There was however no comprehensive mental health plan, although mental health was a key component of the Health Sector Strategic Plan. The programme had no disaster preparedness plan for mental health. However, one of the core interventions in the Health Sector Strategic Plan was addressing mental health in conflict situations.

### Legislation and Human rights protection

The mental health system operates on an outdated mental health law that was last revised in 1964. The legislation focuses on custodial care of mentally ill persons and is not in accordance with contemporary international human rights standards regarding mental health care. This obsolete legislation was found to have a number of shortcomings such as failure to distinguish voluntary and involuntary care, inadequate protection and promotion of the human rights of people with mental illness and the presence of derogatory and stigmatizing language; and was thus not in line with the draft mental health policy as well as current trends in mental health care.

Furthermore, there were no legislative provisions to provide support for users in the following areas:

• a legal obligation for employers to hire a certain percentage of employees that are mentally disabled

• provisions concerning protection from discrimination at work (dismissal, lower wages etc) solely on account of mental disorder; and

• financial provision concerning protection from discrimination in allocation of housing and subsidized housing schemes for people with severe mental disorder

There was no national or regional human rights review body for assessing the human rights protection of users in mental health services. Neither the mental hospital nor in-patient psychiatric units in the general hospitals had arrangements for review of protection of patients' human rights protection. Similarly, neither the mental hospital nor any psychiatric units had specific trainings, meetings or any other type of working sessions on human rights protection of patients. However, some of the health workers had had some general training on human rights issues among the mentally ill as part of their overall training.

### Financing of mental health services

**Figure 1:**
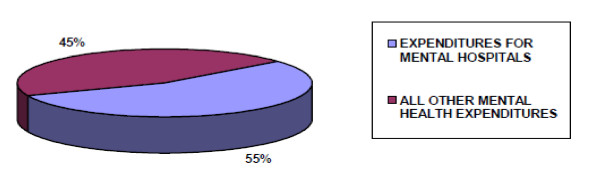
**National expenditure towards mental health**.

About 1% of expenditure by the government health department was directed towards mental health in primary care. However, as part of the integrated health service delivery, other aspects of mental health were funded within the general health budget as well. Furthermore, under donor support to the government, the health sector's financing was at the time supplemented by funding from the African Development Bank (ADB), with nearly 45% of the support going to mental health. This raised the expenditure on mental health to approximately 4% of health care expenditure. Of all the mental health expenditures, 55% was directed towards the National Mental Hospital (Figure 1).

One hundred percent of the population has free access to essential psychotropic medicines. This is based on the fact that medication is provided at no cost in all public health facilities. For those who pay out of pocket, 37% of the daily minimum wage was needed to pay for one day's dose of antipsychotic medication while 7% of daily the wage was needed to pay for one day's dose of antidepressant medication. Mental disorders were not covered in the social insurance schemes, but were listed in the proposed National Health Insurance Scheme.

### Mental health services

#### Organization of mental health services

There was an office for coordination of mental health services at the Ministry of Health headquarters, occupied by a Principal Medical Officer. The main roles for the office included policy development, resource mobilisation, planning and coordination, as well as monitoring and quality assessment of the mental health services in the country. Mental health services to communities were organized on the basis of catchment/service areas at Regional and District levels. The only national mental hospital also offered general health services at the outpatient's facility to the population in the neighbourhood within a radius of 10 Kilometres.

#### Mental health outpatient facilities

There were 28 outpatient mental health facilities available in the country, with none having a special clinic for children and adolescents only. The number of users per 100,000 general population treated by these facilities could not be established. However, they treated a total of approximately 13,710 new users in 2005. Of all the new users treated, 40% were females, while 16% were children and adolescents. The majority of users treated in outpatient facilities were diagnosed with mood disorders and epilepsy, although reliable data on the diagnoses per disorder were not available. The average number of contacts per user could not be established. Fifty four percent of the outpatient facilities provided follow-up care in the community and conducted outreach clinics whenever they had funds, although this was not a routine practice. Only a few of the users (1-20%) had received one or more psychosocial interventions during the previous year. Fifty seven percent of the mental health outpatient facilities had at last one psychotropic drug of each therapeutic class (anti-psychotic, antidepressant, mood stabilizer, anxiolytics, and antiepileptic medicines) available in the facility or a near-by pharmacy all year round.

#### Day treatment facilities

There was only one day treatment facility available in the country. This facility treated 175 users. Of all users treated in day treatment facilities, 49% were females and 36% are children or adolescents.

#### Community-based psychiatric inpatient units

There were 27 community-based psychiatric inpatient units available in the country with a total of 382 beds (1.4 per 100,000 population). These facilities are the psychiatric units in all hospitals other than the National Mental Hospital. Fifteen percent of these beds were reserved for children and adolescents. Aggregated information on admissions and diagnoses in these units was not available. One to twenty percent of patients in community-based psychiatric inpatient units received one or more psychosocial interventions in the previous year. Thirty seven percent of community-based psychiatric inpatient units had at least one psychotropic medicine of each therapeutic class (anti-psychotic, antidepressant, mood stabilizer, anxiolytic, and antiepileptic medicines) available in the facility. There are no community residential facilities available in the country.

#### Mental hospitals

The only mental hospital in the country had both inpatient and outpatient facilities. The hospital had a total of 500 beds (1.83 beds per 100,000 population), the number having increased by 11% in the previous 5 years. No beds in the mental hospital had been reserved specifically for children and adolescents. Of all the patients treated in the mental hospital, 41% were females and 16% children and adolescents. No reliable data on diagnostic groups was available. However, anecdotal data indicated that the patients admitted to the mental hospital were of two main diagnostic groups: mood disorders and epilepsy. The hospital had a bed occupancy rate of 100%. The average number of days spent in the mental hospital could not be established. However, almost all patients spent less than a year in the hospital except for a few mentally-ill offenders. The tendency of patients to escape from hospital was still common; and this partly made it hard to determine the accurate number of days spent in the mental hospital. The number of patients physically restrained or secluded could not be established. Less than half of the patients in mental hospital had received psychosocial interventions in the previous year. The mental hospital had at least one psychotropic medicine of each therapeutic category (antipsychotic, antidepressant, mood stabilizer, anxiolytic and antiepileptic medicines) available all year long.

#### Forensic and other residential facilities

The mental hospital also had a forensic inpatient unit with a bed capacity of 116 beds (0.42 beds per 100,000 general population). However, only about 10% of the beds in this unit were occupied by the mentally-ill offenders. This category was of long-stay patients, with some who had spent more than 5 years in the unit. There were 7 other non-public residential facilities, 4 of these for children and adolescents with mental retardation while 3 were for people with alcohol and substance use problems. There were a total of 120 beds for youths aged 17 years and below with mental retardation and 30 beds for people with substance abuse problems.

#### Distribution of services

Sixty two percent (62.4%) of the psychiatric beds in the country were located in or near the largest city; a distribution pattern that limits access for rural users. On average, there was a substantial difference between government-administered and private for-profit mental health care facilities in terms of the average time for an outpatient consultation with a psychiatrist and average number of beds per nurse in the facility. Inequity of access to mental health services for other minority users (e.g., linguistic, ethnic, religious minorities) was not an issue in the country.

The biggest challenge in determining the number of users treated per facility, diagnoses and other information was the fact that for the few facilities where information was available, the information was based on the number of attendances and not specific patients. The percentage of users for children and/or adolescents varied substantially from facility to facility. The proportion of child users was highest in the day treatment facility and lowest in mental hospital. Psychotropic medication was mostly widely available in the mental hospital.

#### Mental health in primary health care

There were both physician based and non-physician based primary health care (PHC) clinics in the country. For physician-based PHC clinics, a few (1-20%) had assessment and treatment protocols for key mental health conditions available, just like the non-physician-based primary health care clinics. The majority (51-80%) of physician-based primary health care doctors and the non-physician based primary health care clinics made at least one referral per month to a mental health professional on average. Only a few of the PHC doctors had interacted with a mental health professional at least once in the previous year. A few (1-20%) of physician-based PHC facilities have had interaction with a complimentary/alternative/traditional practitioner, in comparison to none of the non-physician based PHC clinics, and a few (1-20%) of the mental health facilities.

While doctors, clinical officers and nurses were allowed to prescribe and/or to continue prescription of psychotropic medicines with restrictions, other PHC workers were not allowed to prescribe psychotropic medications. For example clinical officers and nurses in Primary Health Care cannot initiate a prescription but can continue a prescription or they can initiate a prescription only in emergencies. In contrast, psychiatrists, medical officers and psychiatric clinical officers were allowed to prescribe psychotropic medications without restrictions. As regards the availability of psychotropic medications, only some of the physician based PHC clinics (21-50%) had at least one psychotropic medicine of each therapeutic category (anti-depressant, antipsychotic, mood stabilizer, anxiolytic and anti-epileptic) in comparison to a few (1-20%) of the non-physician based PHC clinics.

About ten percent of the training for medical doctors was devoted to mental health, in comparison to 3% for General Nurses. In terms of refresher training, the proportion of primary health care staff with at least two days of refresher training in mental health could not be established.

#### Information systems in mental health services

There was a formally defined list of specific data items that ought to be collected by all mental health facilities. The extent of data collection was variable among the mental health facilities, though many of the facilities were not collecting the expected data. Both mental hospital and community based psychiatric inpatient units collected and compiled data though there were gaps. Of the data that the mental hospital collected and compiled, only data on number of beds were reliable. Mental heath data were published by the government health department in an annual performance report. All the mental health facilities routinely reported and transmitted data to the government health department, with the lower facilities doing this through their supervising levels.

### Human resources

The total number of human resources working in mental health facilities or private practice per 100,000 population was 1.13, with each category as follows: 0.08 psychiatrists; 0.04 other medical doctors; 0.78 nurses; 0.01 psychologists; 0.01 social workers; 0.01 occupational therapists; and 0.2 psychiatric clinical officers; not including other health care workers such as auxiliary staff, non-doctor PHC workers and health assistants. Five Percent of the psychiatrists worked for only government administered facilities, 5% for only NGOs/for profit mental health facilities/private practice; while 90% worked for both sectors. Accurate data on distribution of the other professionals was not readily available. All the professionals worked for both in and outpatient facilities. Fourteen psychiatrists worked in community based psychiatric inpatient units and 8 in the mental hospital. The 8 other medical doctors mentioned, who are non-mental health specialists all worked in the mental hospital. Sixty two mental health nurses worked in community based psychiatric inpatient units, while 153 work in the mental hospital. Three of the psychosocial staff worked in the community based psychiatric inpatient facilities and the other 3 in the mental hospital. Only 0.8% of all the medical doctors and 4% of all the nurses in the country were specialists in psychiatry.

**Figure 2:**
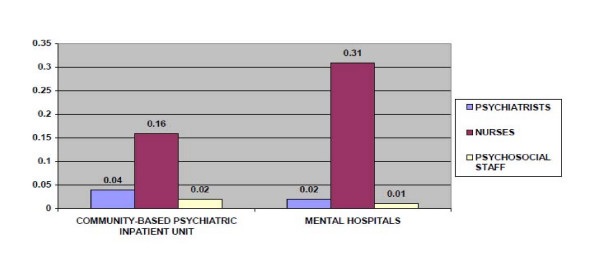
**Average number of staff per bed**.

As for human resources in mental health facilities, there were 0.04 psychiatrists per bed in community based psychiatric inpatient units in comparison to 0.02 psychiatrists per bed in mental hospitals. There were 0.16 nurses per bed in community based psychiatric inpatient units as compared to 0.31 nurses per bed in the mental hospital. Accurate data for other mental health staff were not readily available. The distribution of human resources between the urban and rural areas was disproportionate. The density of psychiatrists in or around the largest city was 11 times greater than the density of psychiatrists in the entire country. The density of nurses was 13.4 times greater in the largest city than the entire country (Figure 2).

The number of professionals who had graduated the previous year in academic and educational institutions was as follows: 162 general medical doctors, 4 psychiatrists, 13 psychologists with at least 1 year training in mental health care, 10 occupational therapists with at least 1 year training in mental health care. The number of general nurses and that of social workers with at least 1 year training in mental health care could not be established. However, there were 1,491 nurses who registered with the nurses and midwives council that year. None of the psychiatrists emigrated to other countries within 5 years of completion of their training. The accurate number of mental health care staff with at least 2 days of refresher training in the rational use of drugs, psychosocial interventions and child and adolescent issues was not readily available.

### Consumer and family associations

There were 2,225 users/consumers who were members of user associations. However, the numbers of families who were members of family associations was unknown. Furthermore, there was no arrangement by government to provide financial support to user associations for mental health initiatives. Consumer associations had been involved in formulation and implementation of the mental health policy and plan to some extent; but not the legislation. Similarly, there were 4 NGOs involved in mental health initiatives, and these had been involved in the mental health development exercise. Very few mental health facilities interacted with consumer/user associations.

### Public education and awareness campaigns on mental health

The Principal Medical Officer in charge of mental health at the Ministry of Health oversees public education and awareness campaigns on mental health and mental disorders. Government agencies, NGOs, professional associations and international agencies had promoted public education and awareness campaigns in the previous 5 years. The campaigns had targeted the general population, children, adolescents, women, and trauma survivors. In addition, there had been public education and awareness campaigns targeting professional groups including: health care providers (conventional, modern, allopathic, complementary/alternative/traditional healers), teachers, social services staff, politicians and other professional groups linked to the health sector.

### Mental health links with other sectors

There were formal collaborations between the government department responsible for mental health and the departments/agencies responsible for primary health care/community health, HIV/AIDS, reproductive health, child and adolescent health, substance abuse, child protection and education. As regards support for child and adolescent mental health, information on the proportion of primary and secondary schools that hade either a part-time or full time mental health professionals was not available. The number of primary and secondary schools with school based activities to promote mental health and prevent mental disorders was unknown. The percentage of prisoners with psychosis was greater than 15% while that with mental retardation was unknown. Only a few (1-20%) of the police officers, judges and lawyers had received training in mental health in the previous 5 years. There was no mental health facility where users had access to programs that provide outside employment and there were no patients reported to have received social welfare benefits for their mental disability.

### Mental health research

Only about 2% of all the health publications in Uganda during the previous 5 years were on mental health. Research had focused on some areas including epidemiological studies in community samples and clinical samples, non-epidemiological clinical/questionnaires assessments of mental disorders, services research and psychosocial/psychotherapeutic interventions. It was noted that the ministry of health recognizes the importance of research to provide evidence-based practice although it is not well funded.

## Discussion

The findings of this study reveal significant success amidst a number of challenges facing Uganda's mental health system. On the one hand, mental health is one of the priority areas within the Ministry of Health's strategic plan, and one of the components of the National Minimum Health Care Package. Furthermore, situated within a public health and primary health care framework, there is a draft mental health policy in place. Such developments suggest significant advancement of the mental health system as compared to many other low and middle-income countries. The WHO estimates that over 40% of developing countries do not have a mental health policy and over 30% have no mental health programmes [17]. Furthermore, guided by this draft policy, a number of reforms have been initiated, which have significantly strengthened the mental health services in the country. These include decentralization of mental health services; integration of mental health into Primary Health Care, establishment of mental health inpatient units within the Regional Referral Hospitals; training of general health workers in mental health (pre-service and in-service training) and the involvement of other players such as Civil Society Organizations, Traditional healers, and other relevant sectors. In particular, Uganda's efforts towards decentralizing mental health services have been met with much success, compared to many other African countries where efforts have been poor or even non-existent [18-21].

On the other hand, there are a number of challenges. Firstly, the progressive mental health care policy is undermined by the outdated and offensive mental health legislation, which fails to protect and promote the human rights of people with mental disorders. Uganda is part of the 15% of countries which have mental health legislations dating back to the pre-1960s [15]. Mental health legislation is necessary for protecting the rights of people with mental disorders, who are a very vulnerable section of society. The widespread abuse that people suffering from mental disorders frequently experience in Uganda, such as violence, stigma, and employment exploitation, appears to be at least partially symptomatic of the absence of an adequate mental health law [22,23].

Secondly, mental health is still significantly underfunded, with only about 1% of health care expenditures by the government directed towards mental health. Consequently, like many other African countries the mental health care sector relies heavily on donor-funding [24]. Although the level of financing generated through this mechanism appears to be relatively high in Uganda, there are many dangers involved with an over-reliance on donor-funding, including its unreliable, unsustainable and sporadic nature [24], as well as the frequently attached conditionalities [25]. In addition, 55% of the funds that are dedicated to mental health are directed towards the National Mental Hospital, resulting in a continued over-reliance on hospital-based, institutionalized care. This ultimately, hinders steps towards integration of mental health care into the PHC at lower levels of care. This is clearly reflected in the fact that the predominant form of inpatient mental health service provision continues to be based in mental hospitals, with for example, no beds available in community residential facilities.

Thirdly, there is widespread inequality between urban and rural areas in the resources available for mental health care, including staffing and inpatient beds. Like in many other low-income African countries, the availability of psychiatric care is significantly skewed in favour of the urban city centres, with an inequitable geographical spread of services [26,27]. For example, the results revealed that 62.4% of the psychiatric beds in the country were located in or near the largest city, Kampala, with limited access for rural users. The situation is further complicated by a striking absence of reliable, routinely collected data that can be used to plan for services and redress current inequalities.

Finally, there is an absence of specialised child and adolescent mental health services in place, with children and adolescents being treated in the same facilities as adults; a common problem in most developing countries [28]. This is despite the fact that children and adolescents constitute over 50% of the country's population [2], and 16% of all new users of mental health outpatient facilities were children and adolescents. The lack of attention afforded to child and adolescent mental health is somewhat surprising, given that the mental health needs of children and adolescents are specifically mentioned in Uganda's draft mental health policy [29]. Furthermore, the Ugandan government has signed and ratified the Convention on the Rights of the Child, which obliges the government to ensure the maximum possible development and best health care for children [30].

This study underscores the need for further research on mental health in Uganda. As indicated by the results from this study, only 2 - 4% of all health publications in Uganda were on mental health. More research is needed to explore the current burden of mental disorders and evaluate the development and functioning of mental health programs and services. This information can then serve as a basis for increased advocacy strategies and investment in mental health.

## Conclusion

Compared to many other low-income African countries, Uganda's mental health care sector has made significant strides forward. The formulation of a new progressive mental health policy, and the implementation of numerous service reforms within the country are testimony to Uganda's increased commitment to meeting the health care needs of the country.

Despite these reforms, and the consequent strengthening and improvement of mental health services, Uganda's mental health system still possesses a number of shortcomings. In this light, a number of policy and service development initiatives are required. Firstly, there is a need for stronger national leadership to finalize and enact the current draft mental health policy. The policy should also better address the mental health care needs of children and adolescents. Secondly, there is a need to review the outdated mental health legislation to bring it up to date with current International Standards. The law should provide a legal framework for protecting the rights of the mentally ill, and addressing critical issues such as the community integration of persons with mental disorders, the provision of care of high quality, the improvement of access to care, the protection of civil rights and the protection and promotion of rights in other critical areas such as housing, education and employment [31]. Thirdly, a nationally agreed minimum data set needs to be put in place and an information system established, in order to consistently monitor mental health service delivery district and national levels.

## Limitations of the study

The main limitation of this study was that some information could not be collected by the WHO-AIMS, due to the absence of reliable sources.

## Competing interests

The authors declare that they have no competing interests.

## Authors' contributions

FK is the Principal Investigator. He participated in the design of the study and data analysis. He is the lead author and has done most of the editorial work of this paper. SJ is the Research Officer on the project. He collected and analyzed data, with input from other colleagues working on the project. He is the corresponding author. DK is a Research Assistant. She was involved in the data collection and analysis; and has contributed in the alignment of the paper. SC is another Research Officer. She conceived the idea of this paper and did the literature search for the background information and sequence of the paper. She has also played an editorial role. SN is a co-investigator on the project. She was greatly involved in the analysis of the data and has also edited the initial drafts of the paper. The listed MHaPP group members conceived this study and were jointly involved in designing the data collection and analysis methods. All authors read and approved the final manuscript.

## Authors' information

FK is a Senior Consultant Psychiatrist and Executive Director of Butabika National Referral Mental Hospital, Uganda. He is the Ugandan Principal Investigator on the Mental Health and Poverty Project. He is also the President Uganda Psychiatric Association, and formerly WPA zone 14 (East and Southern Africa) Representative.

JS is a Clinical Psychologist and is currently a Research Officer on the Mental Health and Poverty Project. He is also a part-time Assistant Lecturer in the Department of Mental Health and Community Psychology, Makerere University.

DK is a Senior Clinical Psychologist at Butabika National Referral Mental Hospital, Uganda. She is currently pursuing Doctoral studies at Norwegian University of Science and Technology.

SC is a Research Officer in the Mental Health and Poverty Project, Department of Psychiatry and Mental Health, University of Cape Town, South Africa. He holds a Masters degree in Public Health from the same University.

SN is a Public Health Specialist and the Principal Medical Officer in charge of mental health at the Ministry of Health headquarters in Uganda.

The Mental Health and Poverty Project (MHaPP) is a Research Programme Consortium (RPC) funded by the UK Department for International Development (DfID)(RPC HD6 2005-2010).
